# Nutritional frailty and sarcopenia risk among older Somali refugee returnees: an overlooked crisis at the intersection of displacement, aging, and food insecurity

**DOI:** 10.1016/j.jnha.2026.100901

**Published:** 2026-06-11

**Authors:** Mohamed Abdulkadir Hussein, Saida Abdirzaak Mohamed, Ilyas Abdullahi Khalif, Salma Zubair Mohamud, Hibo Abdullahi Mohamed

**Affiliations:** a Faculty of Health Sciences and Tropical Medicine, Somali National University, Mogadishu, Somalia; b Center for Health Research and Innovation, Somali National University, Somalia

Older adults in low- and middle-income countries (LMICs) experience a high and growing burden of sarcopenia and nutritional frailty, which are strongly linked to functional decline, disability, and mortality [1]. Severe food insecurity in LMICs more than doubles the odds of sarcopenia among adults aged ≥65 years, underscoring the vulnerability of older people where safety nets are weak. Humanitarian crises further magnify these risks: in Somalia, internally displaced populations face persistently high mortality, with severe malnutrition among the leading causes of death. Yet older refugee returnees, who have survived prolonged displacement and now age in fragile, food-insecure environments, remain largely invisible in epidemiological data and are seldom considered in aging or humanitarian health agendas. Their situation represents a critical blind spot at the intersection of displacement, aging, and food insecurity.

Displacement-related food insecurity exposes older adults to chronic protein–energy malnutrition and inadequate micronutrient intake, which are central drivers of sarcopenia and frailty. Malnutrition and low protein intake are consistently associated with reduced skeletal muscle mass, weaker grip strength, and slower gait in community-dwelling older adults, leading experts to call for routine, integrated screening of nutritional status and sarcopenia in primary care and community settings [3]. At the biological level, sarcopenia is characterized by an imbalance between muscle protein synthesis and breakdown, occurring in the context of age-related immune senescence and chronic low-grade inflammation. Older people with sarcopenia show significantly higher circulating TNF-α, IL-1β, and IL-6 compared with non-sarcopenic peers, and combined resistance exercise with branched-chain amino acid and vitamin D supplementation improves muscle mass and strength while lowering these inflammatory markers. Such data provide a mechanistic basis for how prolonged exposure to undernutrition, micronutrient deficiency, inactivity, and psychosocial stress in refugee settings can accelerate catabolic muscle loss and nutritional frailty (Table 1).

**Table 1:** Key evidence linking food insecurity, malnutrition, and sarcopenia in older adults.

Theme	Key Finding
Food insecurity & sarcopenia	Severe food insecurity is associated with approximately twofold higher odds of sarcopenia
Inflammation & muscle loss	Older adults with sarcopenia show elevated TNF-α, IL1β, and IL6; Improved with combined exercise and nutritional interventions [2].
Malnutrition in Somalia	Severe malnutrition is a major cause of mortality
Nutrition & screening	Malnutrition is closely linked to reduced muscle mass and physical function; combined nutrition–sarcopenia screening is recommended.
Intrinsic capacity & outcomes	Lower intrinsic capacity is associated with greater functional decline and mortality and higher mortality.

Somalia’s protracted crisis illustrates how these mechanisms translate into heightened vulnerability. Recurrent conflict, drought, and food price shocks have produced one of the world’s most complex humanitarian emergencies, with internally displaced persons experiencing high crude death rates and an excess of mortality driven by severe malnutrition and infectious disease [4]. In such contexts, disruptions to traditional, nutrient-dense food systems and constrained access to diverse diets amplify protein and micronutrient deficits in older adults. Evidence from Asian and LMIC community cohorts shows that malnutrition risk is tightly linked to low skeletal muscle mass, weaker handgrip strength, and slower gait speed, reinforcing that undernutrition and sarcopenia co-occur and should be screened together. However, validated tools such as the Mini Nutritional Assessment and simple anthropometric measures (e.g., calf circumference) are seldom embedded in routine services in fragile settings, and there is no indication that sarcopenia-specific instruments like SARC-F are systematically used in humanitarian health programs. This likely renders frailty and sarcopenia among older Somali returnees clinically invisible despite their probable high burden of malnutrition and functional impairment.

The WHO concept of intrinsic capacity—the composite of physical and mental capacities—offers a useful framework to understand the downstream consequences of this neglected problem. Lower intrinsic capacity is consistently associated with greater subsequent impairment in basic and instrumental activities of daily living and with increased mortality risk in older adults, while maintained or improved intrinsic capacity is linked to lower odds of functional decline. Since nutrition, muscle strength, and mobility form core domains within intrinsic capacity, unaddressed nutritional frailty and sarcopenia in older returnees are likely to precipitate cascading declines in function, dependency, and healthspan [5]. Given that sarcopenia is also associated with higher risks of falls, hospitalization, and poor mental health, failure to intervene may lead to accelerated biological aging and premature loss of independence in a population already burdened by the cumulative insults of displacement and adversity. These trajectories sit squarely within the Journal of Nutrition, Health & Aging’s mission to promote nutrition-driven healthy longevity across the life course.

Taken together, current evidence supports a strong, biologically plausible linkage between food insecurity, malnutrition, inflammation, and sarcopenia in older adults in LMICs and humanitarian settings, and shows that declines in intrinsic capacity are tightly coupled with functional loss and mortality. What remains largely unmapped is how these processes unfold specifically among older Somali refugee returnees, who transition from camp-based deprivation to fragile, food-insecure communities with minimal geriatric or nutritional services. There is an urgent need to explicitly include older returnees in national and humanitarian nutritional surveillance, to adapt and validate brief tools such as SARC-F and the Mini Nutritional Assessment for Somali use, and to incorporate low-cost anthropometric measures into primary care and camp-to-community reintegration packages. Given the demonstrated benefits of combining resistance exercise with protein- and micronutrient-rich supplementation on muscle mass, strength, and inflammatory status in sarcopenic older adults, targeted programs for this group are both scientifically justified and potentially transformative. Addressing nutritional frailty and sarcopenia among older Somali returnees is therefore not only a clinical and public health imperative, but also a moral one, aligning directly with global commitments to healthy aging and leaving no one behind.

## CRediT authorship contribution statement

MAH conceptualized the manuscript topic and framework. SAM and SZM contributed to literature review and drafting of the manuscript. IAK and HAM contributed to critical revision and editing. All authors reviewed and approved the final manuscript.

MAH conceptualized the study and designed the manuscript framework. SAM and SZM performed the methodology, conducted the formal analysis, and drafted the original manuscript. IAK and HAM directed the review and editing process, developed the visualizations, and contributed substantially to manuscript drafting. All authors critically evaluated the work for scientific accuracy and rigor, and approved the final version for submission.

## Consent for publication

Not applicable.

## Ethics approval and consent to participate

Not applicable. This is based exclusively on previously published literature and does not involve human participants or primary data collection.

## Declaration of Generative AI and AI-assisted technologies in the writing process

The authors used ChatGPT to assist with language editing and manuscript organization. All authors reviewed and approved the final manuscript and take full responsibility for the content.

## Funding

This research received no external funding.

## Availability of data and materials

Not applicable.

## Clinical trial number

Not applicable.

All authors have reviewed and approved this declaration.

## Declaration of competing interest

The authors declare no competing interests.
